# Transcriptome Analysis of a Bloom-Forming Cyanobacterium *Microcystis aeruginosa* during Ma-LMM01 Phage Infection

**DOI:** 10.3389/fmicb.2018.00002

**Published:** 2018-01-19

**Authors:** Daichi Morimoto, Shigeko Kimura, Yoshihiko Sako, Takashi Yoshida

**Affiliations:** ^1^Laboratory of Marine Microbiology, Graduate School of Agriculture, Kyoto University, Kyoto, Japan; ^2^School of Environmental Science, University of Shiga Prefecture, Hikone, Japan

**Keywords:** *Microcystis aeruginosa*, cyanophage, Ma-LMM01, RNA sequencing, toxic bloom

## Abstract

*Microcystis aeruginosa* forms massive blooms in eutrophic freshwaters, where it is constantly exposed to lytic cyanophages. Unlike other marine cyanobacteria, *M. aeruginosa* possess remarkably abundant and diverse potential antiviral defense genes. Interestingly, T4-like cyanophage Ma-LMM01, which is the sole cultured lytic cyanophage infecting *M. aeruginosa*, lacks the host-derived genes involved in maintaining host photosynthesis and directing host metabolism that are abundant in other marine cyanophages. Based on genomic comparisons with closely related cyanobacteria and their phages, Ma-LMM01 is predicted to employ a novel infection program that differs from that of other marine cyanophages. Here, we used RNA-seq technology and *in silico* analysis to examine transcriptional dynamics during Ma-LMM01 infection to reveal host transcriptional responses to phage infection, and to elucidate the infection program used by Ma-LMM01 to avoid the highly abundant host defense systems. Phage-derived reads increased only slightly at 1 h post-infection, but significantly increased from 16% of total cellular reads at 3 h post-infection to 33% of all reads by 6 h post-infection. Strikingly, almost none of the host genes (0.17%) showed a significant change in expression during infection. However, like other lytic dsDNA phages, including marine cyanophages, phage gene dynamics revealed three expression classes: early (host-takeover), middle (replication), and late (virion morphogenesis). The early genes were concentrated in a single ∼5.8-kb window spanning 10 open reading frames (gp054–gp063) on the phage genome. None of the early genes showed homology to the early genes of other T4-like phages, including known marine cyanophages. Bacterial RNA polymerase (σ^70^) recognition sequences were also found in the upstream region of middle and late genes, whereas phage-specific motifs were not found. Our findings suggest that unlike other known T4-like phages, Ma-LMM01 achieves three sequential gene expression patterns with no change in host promoter activity. This type of infection that does not cause significant change in host transcriptional levels may be advantageous in allowing Ma-LMM01 to escape host defense systems while maintaining host photosynthesis.

## Introduction

Viruses are extremely abundant in aquatic environments, with global estimates reaching 10^30^ virus-like particles (Suttle, 2005). Viruses are thought to play important roles in regulating the abundance, clonal diversity, and composition of bacterial populations (Suttle, 2007), and thus have the potential to affect biogeochemical cycles through the process of host cell lysis (Suttle, 2005, 2007). Therefore, it is essential to elucidate viral infection mechanisms to better understand the impact of viruses on host populations and biogeochemical cycles.

In general, infection dynamics of T4-like phages show that following infection, host genomic DNA is degraded and there is an almost complete shift to phage transcription, leading to the shutdown of host metabolism (Miller et al., 2003; Roucourt and Lavigne, 2009). The phage transcriptional program generally follows the three temporal expression classes of early, middle, and late genes, which correspond to host takeover, replication, and virion morphogenesis, respectively (Luke et al., 2002; Roucourt and Lavigne, 2009). In T4 phage, this expression program is regulated by the sequential modification of the host RNA polymerase and associated σ factor, leading to consecutive changes in affinity for different promoter sequences. The expression of early genes relies on the primary host σ^70^ factor which recognizes early T4 promoters that resemble the major *Escherichia coli* promoters and is stronger than any bacterial promoters (Miller et al., 2003). The internal head protein Alt increases affinity for the early T4 promoters and supports preferential transcription from early T4 promoter by ADP-ribosylation of one of the two α subunits of the host RNA polymerase (Rohrer et al., 1975; Goff and Setzer, 1980). Middle-gene promoters have a distinctive motif sequence that is again recognized by the host σ^70^ factor, aided by phage-encoded proteins AsiA and MotA. Anti-σ factor AsiA forms the heterodimers with host σ^70^ factor, and activates the transcription from middle T4 promoters. Transcriptional activator MotA binds to the MotA box sequence and recruits the host RNA polymerase to middle T4 promoters (Marshall et al., 1999; Sharma et al., 1999). Phage-encoded proteins endoribonuclease RegB (Sanson et al., 2000) and ADP-ribosyltransferase ModA (Tiemann et al., 2004) also contribute to switch from the early transcription to middle transcription. In contrast to transcription from early and middle-gene promoters, recognition of late-gene promoters requires a phage-encoded σ factor, gp55. Co-activator gp33 and DNA-loaded sliding clamp gp45 also involve in efficient transcription from late-gene promoters (Roucourt and Lavigne, 2009).

T4-like cyanophages infecting marine cyanobacterial genera *Synechococcus* and *Prochlorococcus* contain homologs of the T4 replication and virion structural genes that are shared among T4-like phages (T4 core genes) (Sullivan et al., 2010). According to their genomic features, transcriptome analyses for marine T4-like cyanophages clearly indicate the three temporal classes of early, middle, and late genes as seen in T4 phage (Clokie et al., 2006; Doron et al., 2016; Lin et al., 2016; Thompson et al., 2016). In addition, marine T4-like cyanophages possess a number of auxiliary metabolic genes (AMGs) that are derived from hosts and are involved in processes such as photosynthesis, carbon metabolism, and phosphorus utilization (Mann et al., 2005; Thompson L.R. et al., 2011). Such AMGs are thought to provide support during key steps in host metabolism that are relevant to phage, thereby boosting and redirecting host metabolism after the shutoff of host metabolism caused by phage infection (Thompson A.W. et al., 2011; Thompson et al., 2016). Indeed, pentose phosphate pathway are augmented to provide a more direct mechanism for NADPH production, where NADPH is not destined for carbon fixation, but rather both ATP and NADPH are available for nucleotide synthesis for viral genome replication during cyanophage P-HM2 infection (Thompson et al., 2016). In this way, T4-like cyanophages maintain host photosynthesis activity and redirect carbon flux from the Calvin cycle to the pentose phosphate pathway, although they lack T4-like middle-gene promoters and the *motA* and/or *asiA* genes (Doron et al., 2016; Thompson et al., 2016).

Toxic bloom-forming cyanobacterium *Microcystis aeruginosa*, along with its phages, provides an excellent model to study the co-evolution of viruses and their hosts (Kuno et al., 2014) because it contains the largest number of defense genes out of all studied bacteria and archaea (Makarova et al., 2011), and is frequently exposed to phage infection (Kuno et al., 2012; Kimura et al., 2013). Cyanophage Ma-LMM01, which is known to only infect *M. aeruginosa* strain NIES-298 among tested strains, is a member of the *Myoviridae* family (Yoshida et al., 2006) and is phylogenetically distinct from other known marine T4-like cyanophages (Yoshida et al., 2008). Coincidentally, Ma-LMM01 lacks almost all of the T4 core genes involved in appropriating host metabolic machinery, replicating the viral genome during infection, and building viral particles (Yoshida et al., 2008). In particular, it does not contain any homologs of phage-encoded σ factor gp55 and transcription factor gp33, both of which are required for late-gene transcription in T4-like phage (Roucourt and Lavigne, 2009). In addition, Ma-LMM01 contains none of the AMGs usually carried by marine cyanophages. These findings indicate that Ma-LMM01 may employ an infection program that differs from that of other marine cyanophages. Ma-LMM01 does possess a homolog of *nblA*, which plays a central role in the degradation of phycobilisomes (Yoshida et al., 2008). The phage-encoded NblA is predicted to be involved in maintaining host photosynthesis (Yoshida et al., 2008; Gao et al., 2012; Yoshida-Takashima et al., 2012). Furthermore, we previously reported that there was no difference in the level of host *psbA* transcription during Ma-LMM01 infection and that levels of genes involved in the Calvin cycle and pentose phosphate pathway also did not change, or were slightly decreased (Honda et al., 2014). Together, these findings suggested that Ma-LMM01 maintains host photosynthesis activity and carbon metabolism by protecting photosystem II using phage-encoded *nblA*. However, little is currently known about whole host transcriptional responses to phage infection, and the infection program employed by Ma-LMM01 to avoid the highly abundant host defense systems during infection.

In this study, we investigated the infection process and transcriptional program of Ma-LMM01 during infection of its sole host, *M. aeruginosa* NIES-298, and assessed host transcriptional responses to infection using RNA sequencing (RNA-seq) analysis.

## Materials and Methods

### Bacterial and Phage Culture Conditions and Experimental Design

*Microcystis aeruginosa* NIES-298 was obtained from the Natural Institute for Environmental Studies (NIES, Japan^1^). *M. aeruginosa* was cultured in CB medium (Kasai et al., 2004) under a 12/12-h light/dark photocycle (light intensity: 21 μmol photons/m^2^/s) at 30°C with 0.5% CO_2_ (v/v) aeration.

To prepare the phage lysate, a 1-L *M. aeruginosa* culture (9.14 × 10^5^ cells/mL) was infected with Ma-LMM01 at a multiplicity of infection of 0.02 and then incubated as above for 3 days. The resultant lysate was filtered through a sterile 3.0-μm polycarbonate membrane filter (Millipore, Billerica, MA, United States) and stored as an original lysate at 4°C (Honda et al., 2014).

A 9-L volume of *M. aeruginosa* culture in exponential-phase was prepared as described above and then divided between six different flasks containing 1.5 L of medium. We performed the one-step growth experiment for Ma-LMM01 to obtain simultaneously infected cells without multiple infection for RNA-seq analysis according to our previous study (Yoshida et al., 2006). Using this infection experiment, at least two different temporal classes (early and late genes) have been observed in expression of phage genes (Yoshida-Takashima et al., 2012). In brief, cell division is well synchronized compared with untreated cells when *Microcystis* cells arrested once by 36 h darkness are transferred to continuous illumination (the block-released method) (Yoshida T. et al., 2005; Yoshida-Takashima et al., 2012). Thereby the variation of infection stage was minimized at each time point. For the phage infected cultures, 250 mL of original phage lysate were added to each of three synchronized cultures after the light was turned on. In parallel with infection experiment, infectious phage concentration was determined using the most probable number (MPN) method (3.59 × 10^6^ infectious units/mL) (Yoshida et al., 2006; Honda et al., 2014), resulting in a multiplicity of infection (MOI) of 0.62–0.89. From the wide range of upper value and lower value within the confidence limits (95%) in MPN method (Wyshak and Detre, 1972), the MOIs varied. However, almost complete lysis was observed at 24 h after infection. In addition, growth experiments of Ma-LMM01 should be carried out with MOIs less than 1 because an MOIs greater than 2 results in a small decrease in the number of host cells (Yoshida et al., 2006). For the control cultures, an equivalent volume of CB medium was added to a further three flasks in place of the phage lysate. To determine the number of phage particles and host cells, samples were collected from the flasks at different time points during the lytic cycle (0, 0.5, 1, 2, 3, 4, 5, 6, 8, 10, 12, and 24 h after phage addition). To enumerate phage particles, samples were passed through a 3.0-μm PTFE membrane filter and then immediately fixed in 20% glutaraldehyde at a final concentration of 1% and stored at 4°C until analysis. To enumerate host cells, cells were immediately fixed in 20% glutaraldehyde at a final concentration of 1% and stored at 4°C until analysis. Densities of the host cells and phage particles were measured using epifluorescence microscopy (Nicon ECLIPSE E800; Nicon, Tokyo, Japan) with SYBR Gold staining (Molecular Probes, Eugene, OR, United States). As described in the previous studies, the estimated latent period of Ma-LMM01 is 6–12 h (Yoshida et al., 2006) and host transcriptional profiles do not show remarkable change between 6 h and 8 h post-infection (Yoshida-Takashima et al., 2012). For RNA extraction, therefore, 100-mL aliquots of the infected and control cultures were collected at 0, 1, 3, and 6 h post-infection, and cells were collected on 3.0-μm PTFE membrane filters. The cells were then resuspended in 5 mL of stop solution (phenol:ethanol, 5:95 v/v) and stored at -80°C (Yoshida T. et al., 2005). At each time point, these procedures were complete within 20 min (Stewart, 2013).

### Sequencing and Analysis of the *M. aeruginosa* NIES-298 Genome

Genomic DNA extraction from *M. aeruginosa* cells was performed using a combination of the potassium xanthogenate-sodium dodecyl sulfate and phenol/chloroform/isoamyl alcohol procedures, as described previously (Tillett and Neilan, 2000; Yoshida M. et al., 2005). The extracted DNA was sheared using a Covaris M220 focused-ultrasonicator (Covaris, Woburn, MA, United States) to an average size of 300 bp. A mate pair library was then prepared using a Nextera Mate Pair Library Prep Kit (Illumina, San Diego, CA, United States) according to the manufacturer’s instructions. The mate pair library was sequenced using a MiSeq Reagent Kit v3 (2 × 150-bp read length; Illumina) and the Illumina MiSeq platform, and assembled using SPAdes ver.3.7.0 (Bankevich et al., 2012). Open reading frames (ORFs) were predicted using GenemarkS (Besemer et al., 2001), and predicted ORFs were annotated by blastp analysis against the National Center for Biotechnology Information (NCBI) non-redundant database (nr) (*E*-value thresholds of < 1e^-3^).

### RNA-Seq Library Preparation for Illumina Sequencing

Total RNA was extracted from 2 mL of the stored cell suspension as described previously (Yoshida T. et al., 2005). The total RNA concentration was measured using a Qubit Fluorometer (Life Technologies, Paisley, United Kingdom) according to the manufacturer’s instructions. RNA integrity was also verified by gel electrophoresis. DNA was removed using TURBO DNase (Ambion, Austin, TX, United States). Genomic DNA depletion was checked to eliminate the effects of DNA contamination on the following RNA-seq analysis using RT-PCR assay and gel electrophoresis with DNA-depleted RNA samples as non-reverse transcribed control (data not shown). For depletion of ribosomal RNA, a Ribo-Zero rRNA removal kit (Bacteria) (Epicentre, Madison, WI, United States) was used according to the kit instructions, and rRNA depletion was verified by Agilent 2100 bioanalyzer (Agilent Technologies, Palo Alto, CA, United States). The rRNA-depleted RNA was then purified using Agencourt RNAClean XP beads (Beckman Coulter Genomics, Danvers, MA, United States) according to the manufacturer’s instructions. The purified RNA was converted to double stranded cDNA using a PrimeScript Double Stranded cDNA Synthesis Kit (TaKaRa Bio, Otsu, Japan), and cDNA libraries (not strand-specific) were prepared using a Nextera XT DNA sample preparation Kit (Illumina). RNA-seq libraries were sequenced using a MiSeq Reagent Kit v3 (2 × 75 bp read length; Illumina) and the Illumina MiSeq platform.

### Mapping and Counting RNA-Seq Reads

Reads from each library were aligned separately to the merged reference genome (Ma-LMM01 plus *M. aeruginosa* NIES-298) using bowtie2 (Langmead and Salzberg, 2012) with option “–score-min L,0,-0.6”. Host 16S and 23S rRNA reads were removed manually from the total reads prior to read mapping. An average of 1 million reads were recovered from each cDNA library at 0, 1, 3, and 6 h post-infection (**Supplementary Table S1**). Rarefaction curves and chao1 indices for each host and viral reads were separately constructed using PAST ver.3.17 (Hammer et al., 2001). Reads from the whole transcriptome library were counted for each gene. Host and viral transcript counts were each normalized as FPKM (fragments per kilobases of exon per million mapped reads). The reads mapping to viral genome at each time point were visualized independently with Integrative Genomics Viewer (Robinson et al., 2011).

### Identification of Differentially Expressed Genes

Differentially expressed host transcripts were identified using the R package DESeq (Anders and Huber, 2010) with blind method for estimateDispersions and DESeq2 (Love et al., 2014) in Bioconductor (Gentleman et al., 2004). This analysis was conducted for the total host reads, considering the global depletion of all host transcripts relative to the total transcript population due to the influx of viral transcripts. Transcript abundances were analyzed separately at each time point, comparing the infected and uninfected treatments. An adjusted *P*-value (*P*-value with a multiple-test correction) < 0.05 indicated a significant difference. In DESeq2, the dispersion estimation procedure replaces the different methods from the DESeq, and treats the samples as replicates for the purpose of dispersion estimation. Due to the differences of dispersion estimation procedures, DESeq analyses with blind method for estimateDispersions detected differentially expressed genes (DEGs), while DESeq2 analyses could not detect any DEGs. We also investigated whether DEGs with unknown function showed similarity to any of the defense islands in the genome of *M. aeruginosa* NIES-843 (GenBank accession no. NC_010296) using a blastn search with default parameters.

### Clustering of Phage Gene Expression

Cluster analysis of Ma-LMM01 gene expression patterns was performed for the normalized and log_2_-transformed transcript levels of the phage genes derived from the RNA-seq data. Hierarchical clustering was performed using Euclidean distance and average linkage metrics as implemented in the R package “stats”. The Jaccard coefficient was used to assess the quality and stability of the number of clusters obtained from hierarchical clustering (Doron et al., 2016). The Jaccard coefficient provides a measure for the similarity of two different sets of clusters, and ranges from zero (dissimilar) to one (similar). For statistical evaluation of the clustering stability, random subsets of the samples containing 70% of the genes were repeatedly (1000 replicates) selected and clustered, and then the Jaccard coefficient was calculated. This procedure was performed with varying numbers of clusters (*k* = 2–7), and the distribution of the Jaccard coefficients obtained was displayed as a histogram. The benhur function in the R/Bioconductor package “clusterStab” was used to carry out the clustering and calculation. The dendrogram of Ma-LMM01 expression clusters was plotted using the dendrogram function (hclust; R package stats). The heat map analyses for viral gene expression profile were conducted using heatmap.2 function in the R/Bioconductor package “gplots”.

### Computational Identification of Promoter Motifs

The upstream regions of all phage genes (300 bp) were collected from the Ma-LMM01 genome. The primary sigma factor recognition sequences were predicted using BPROM^2^ with default parameters. The promoter sequences were aligned separately for each motif (-10 box and -35 box) using ClustalW (Larkin et al., 2007). Logos were prepared using weblogo (Crooks et al., 2004).

### Verification of RNA-Seq Results

RNA-seq results were verified using quantitative reverse-transcription polymerase chain reaction (qRT-PCR) analysis of host (*sigA, rnpB*) and phage genes (*gp005, gp054, gp062, gp087, gp091* and *gp134*) during Ma-LMM01 infection. Total RNA derived from the same samples was reverse transcribed with random hexamers using a SuperScript III First-Strand Synthesis System (Invitrogen, Carlsbad, CA, United States) as per the manufacturer’s instructions. cDNA copies were quantified using SYBR Premix Ex Taq (Tli RNaseH Plus; TaKaRa Bio) with 5 pmol of each of the forward and reverse primers as described previously (Honda et al., 2014). We designed novel primer sets for *gp054* (ATGCCGAACTAAGAAGCCCACGG and CACTTGCTTCACTCGCTGCTCG), *gp062* (GGTGAACCCATCGTGAATGTGCCA and AAGATTTGGGCAACGGCATCACC), *gp087* (GGGATCCGCTAGCGCAGCTG and AGGCGCACGCCAGAAGGAAC) and *gp134* (ATGCTCCTCCTGGTGGTC and ATAGTAATCCTCGCCGTCC). Transcript levels for each gene were normalized to host *rnpB* transcript levels (Yoshida T. et al., 2005).

### Public Data

The genome sequence of *M. aeruginosa* NIES-298 was deposited in the DNA Data Bank of Japan (DDBJ) Mass Submission System (MSS) under the accession numbers BEIU01000001–BEIU01000088. The mRNA expression data were deposited in the DDBJ Sequence Read Archive (DRA) under accession numbers DRR101368–DRR101375.

## Results

### Phage Infection and Transcriptome Dynamics

**FIGURE 1:**
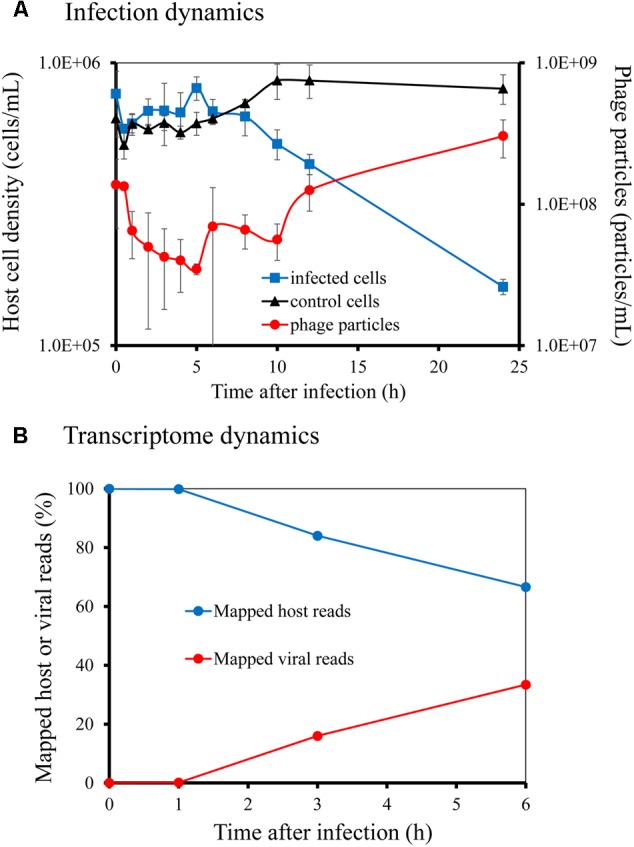
Infection dynamics and transcriptome dynamics of *Microcystis aeruginosa* NIES-298 by myovirus Ma-LMM01. Host cell density and phage particle number were determined by direct count using microscopy with SYBR Gold **(A)**. Ratios of phage and host mRNA at different time points following infection were determined from RNA-seq reads that mapped to the phage and host genomes, respectively **(B)**.

We first investigated the infection process and transcriptome dynamics of the Ma-LMM01 phage during infection. Phage particles were released from the infected cells within 8–12 h of infection (**Figure 1A**), and the number of phage particles increased from 1.38 × 10^8^ particles/mL at 0 h to 3.34 × 10^8^ particles/mL at 24 h post-infection (**Figure 1A**). This infection profile was consistent with previously reported results (Yoshida et al., 2006). In the control culture, the *M. aeruginosa* cell density increased from 6.36 × 10^5^ cells/mL at 0 h to 8.11 × 10^5^ cells/mL at 24 h post-infection (**Figure 1A**). In contrast, in the infected culture, *Microcystis* cells were lysed at the point of phage particle release (**Figure 1A**), with a corresponding decrease in *M. aeruginosa* cell density from 7.78 × 10^5^ cells/mL at 0 h to 1.61 × 10^5^ cells/mL at 24 h post-infection (**Figure 1A**). Considering that Ma-LMM01 infection only occurs in a light cycle (Kimura et al., 2012) and the latent period of this phage is 6–12 h (Yoshida et al., 2006), the decrease in host cell number between 0 h and 24 h post-infection represented lysis dynamics of infected host cells without multiple-infection. Therefore, we calculated that >79% of host cells were finally infected by Ma-LMM01 in the infection experiment. Also, Ma-LMM01 infection was thought to occur within 1 h post-infection according to transcriptome dynamics as described below.

Rarefaction analyses for the number of host ORFs clearly demonstrated that each sequencing data was exhaustive to describe the transcriptional profile (**Supplementary Figure S1**). Rarefaction curves generated from viral reads at 0 and 1 h post-infection did not reach an asymptote, and then finally reached at 3 and 6 h post-infection with the progress of viral infection (**Supplementary Figure S1**). The chao1 indices for each library supported these results (**Supplementary Figure S1**). At 1 h post-infection, phage transcripts inside the infected cell accounted for 0.13% of total cellular transcription, but by 3 and 6 h post-infection, phage transcripts constituted 16 and 33% of total cellular transcription, respectively (**Figure 1B**). Therefore, even at 6 h post-infection, host transcripts still accounted for 67% of cellular transcription. Also, qRT-PCR analyses normalized to host *rnpB* transcript levels (**Supplementary Figure S2**) showed that *sigA* transcription did not change until 8 h post-infection and that *gp091* transcription increased gradually and reached peak levels within 6–8 h post-infection, indicating that the transcript profiles of host and phage genes were well represented by the RNA-seq data (see below) (**Supplementary Figure S3**).

### Host Transcriptional Responses to Phage Infection

**FIGURE 2:**
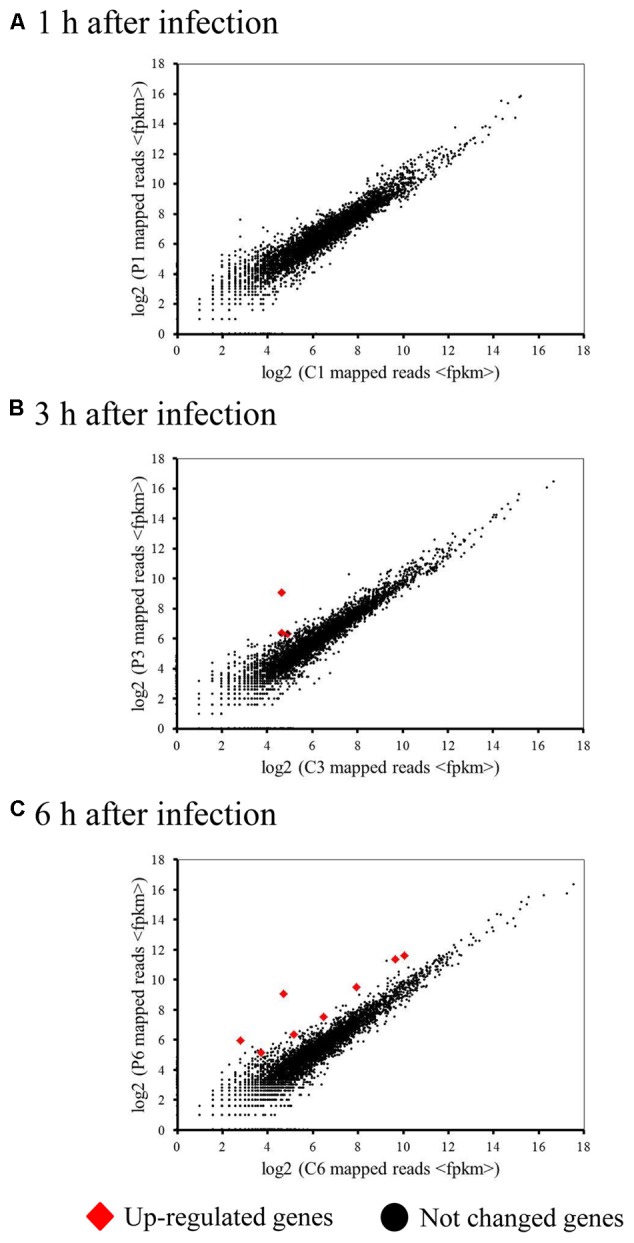
Impact of phage infection on the bacterial transcriptome. Scatter plot of the *Microcystis aeruginosa* transcriptome following phage infection (P1–P6) compared with the uninfected control (C1–C6) at 1 h (early; **A**), 3 h (middle; **B**), 6 h (late; **C**) post-infection. Each dot represents an open reading frame, with up-regulated genes shown in red and unchanged genes in black.

Because a complete switch to phage transcription did not occur by 6 h post-infection, we investigated host transcriptional responses to phage infection in the infected cells. We generated a 4.92-Mb draft genome sequence for *M. aeruginosa* in 88 contigs (≥500 bp), containing a predicted 4749 ORFs. Strikingly, very few (0.17%) of the host genes showed significant changes in expression during infection (**Figure 2**). However, 8 differentially expressed genes (DEGs) were identified during Ma-LMM01 infection although an immediate response was not observed (**Table 1**). Of these, three genes coding for hypothetical proteins were up-regulated after both 3 and 6 h of Ma-LMM01 infection (**Figure 2** and **Supplementary Table S2**). Also, the type I-D CRISPR-associated protein Cas10d/Csc3 gene, membrane protein gene, and three heat shock genes (coding for co-chaperone GroES, molecular chaperone GroEL, and heat-shock protein) were upregulated after 6 h of Ma-LMM01 infection (**Figure 2** and **Supplementary Table S2**).

**Table 1:** Summary of the protein-coding host response genes.

	NIES-298 total in genome		
	1 h	3 h	6 h
Up-regulated genes	0	3	8
Down-regulated genes	0	0	0
Unchanged genes	4749	4746	4741
Total genes	4749		

Amongst the DEGs with annotated function, only the type I-D CRISPR-associated protein Cas10d/Csc3 gene were associated with host defense. However, bacterial genes of unknown function in genomic islands are often differentially expressed in response to viral infection (Lindell et al., 2007; Doron et al., 2016), as well as in response to environmental stressors (Coleman et al., 2006; Thompson A.W. et al., 2011; Stuart et al., 2013; Tetu et al., 2013). Therefore, we next explored where the DEGs with unknown function were located in the *M. aeruginosa* genome. This analysis revealed that three DEGs (identified at 3 h and 6 h post-infection) showed no similarity to genes found in the *M. aeruginosa* NIES-843 defense islands (Makarova et al., 2011). Therefore, three hypothetical protein genes might respond to not phage infection as host defense systems but various stresses for viral production.

### Phage Temporal Expression Patterns

**FIGURE 3:**
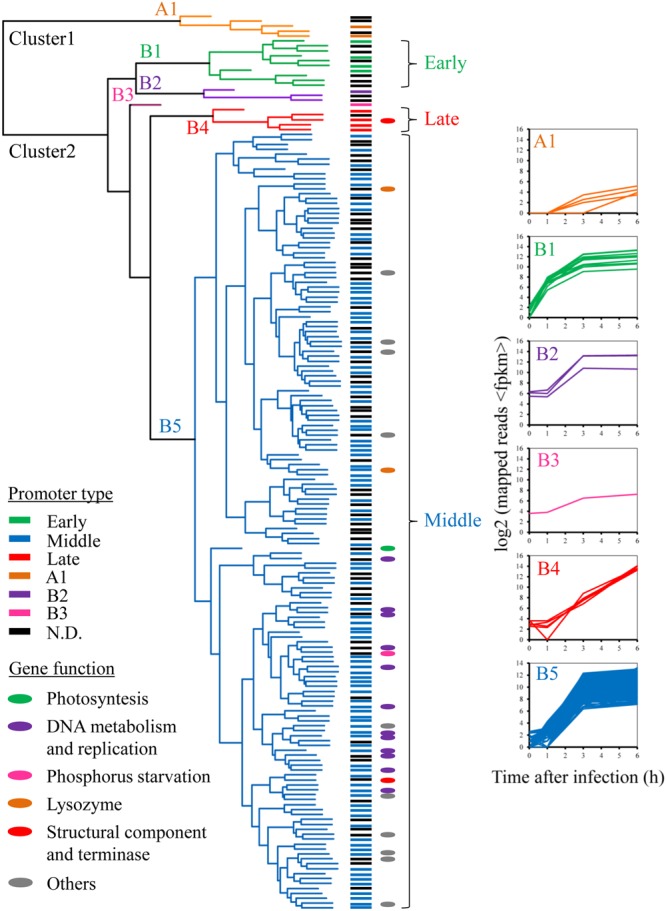
Temporal expression pattern of Ma-LMM01 phage genes. Clustering analysis of phage genes by their expression patterns is presented in the dendrogram, with the A1, B1, B2, B3, B4, and B5 expression subclasses shown in orange, green, purple, pink, red, and blue, respectively. Gene names at the dendrogram tips are colored according to the promoter class driving their expression (see legend, bottom left). The colors of the ovals adjacent to the genes denote the major classes of gene functions (see legend, bottom left). Graphs at the right of the subclasses show expression profiles of the individual genes in that subclasses as a function of time after infection. Subclass designation at the (top left) corner of each graph is as in **Supplementary Table S3**.

Although there was very little change in host gene expression during the course of phage infection, the phage temporal expression classes were apparent when the genes were clustered according to their expression patterns (**Figure 3** and **Supplementary Figure S4**). We assessed the quality and gene composition of the clusters obtained by hierarchical clustering analysis, using the Jaccard coefficient as a stability measure. The most stable solutions were obtained when the phage gene expression profiles were divided into two clusters, with a high frequency of high Jaccard coefficients for this number of clusters (**Supplementary Figure S5**). Cluster 1 was composed of five genes concentrated in a single ∼3.5-kb window in the Ma-LMM01 genome (*gp037–gp041*; **Figure 3**). Cluster 2 comprised 177 genes and could be further divided into five subclasses displaying a variety of expression patterns (B1–B5; **Figure 3**). For example, the expression of subclass B1 genes, which constitute 3.5% of the genome, increased drastically at 1 h post-infection, and then remained high across the remaining sampling points (**Figure 3** and **Supplementary Table S3**). These early genes, spanning 10 ORFs (Gp054–Gp063), were concentrated in a single ∼5.8-kb window in the phage genome (**Supplementary Figure S6**). However, none of the early genes appeared to have homologs in the databases, and showed no homology to early genes of T4 phage and other cyanophages.

All genes in subclass B5 were highly expressed at 3 h post-infection, and expression levels remained high throughout the infection process (**Figure 3**). We identified 162 genes in this middle phase of gene expression, including those involved in DNA replication, recombination/repair, and nucleotide metabolism, as well as genes coding for lysozyme and phage DNA terminase. These included DNA primase (*gp134*), DNA polymerase I (*gp178*), holB-like ATPase (*gp169*), and 3′-5′ exonuclease (*gp180*), all of which are required for phage DNA replication. In addition, the B5 subclass contained genes coding for a T4 RNA-DNA helicase UvsW homolog (*gp166*), RecA-like recombinase UvsX (*gp008*), ATP-dependent RecD-like helicase (*gp160*), and uracil-DNA glycosylase (*gp173*). These four genes are putatively associated with DNA recombination and repair functions. Further middle genes included the α (*gp006*) and β (*gp002*) subunits of ribonucleotide reductase, a flavin-dependent thymidylate synthase ThyX homolog (*gp020*), and dUTPase (*gp181*), all of which are involved in nucleotide metabolism. In addition, a phage-encoded lysozyme and terminase, which are required for cell lysis and DNA packaging, belonged to the middle cluster. The expression patterns of subclasses B2 and B3 were also similar to that of subclass B5 (**Figure 3** and **Supplementary Table S3**).

The expression of all genes in subclass B4 increased gradually during infection, and reached peak levels at 6 h post-infection (**Figure 3**). Overall, we identified five late genes, including those coding for two major head proteins (*gp086* and *gp087*) and a phage tail sheath protein (*gp091*) (Yoshida et al., 2008; **Supplementary Table S3**). This result is consistent with our current understanding of the construction of T4-like phage particles, because phage structural genes tend to be transcribed later in the infection process (Luke et al., 2002; Miller et al., 2003; Doron et al., 2016).

### Viral Regulation of the Transcriptional Patterns

**FIGURE 4:**
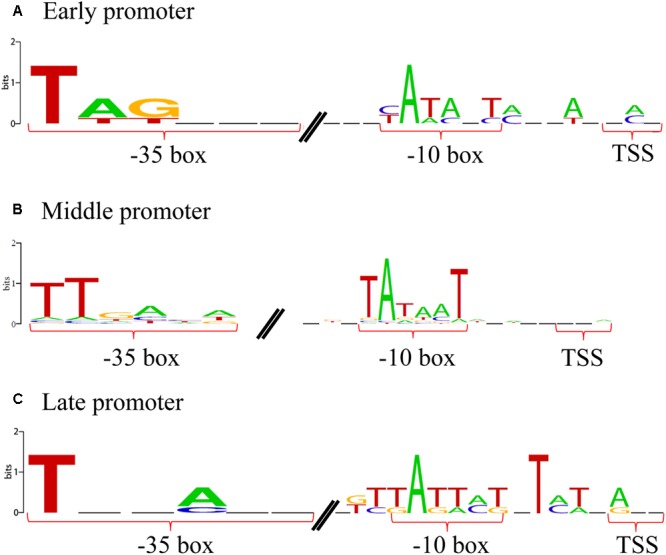
Ma-LMM01 early, middle, and late promoters. Promoter logos of the sequences upstream of the transcription start sites of the phage early **(A)**, middle **(B)**, and late **(C)** genes. Promoter logos were generated from four early gene promoters **(A)**, 97 middle-gene promoters **(B)**, and four late-gene promoters **(C)**.

**FIGURE 5:**
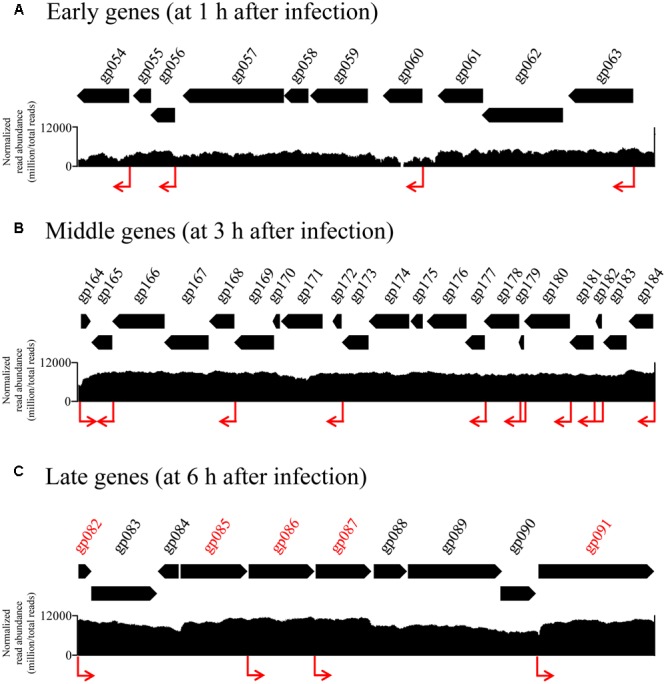
Transcriptomic read mapping pattern of early, middle, and late genes. Part of genome-wide overview of reads mapped to the Ma-LMM01 genome at samples taken 1 h **(A)**, 3 h **(B)**, and 6 h **(C)** after infection, which are composed of early, middle, late genes respectively. Genes shown in red represent the viral late genes **(C)**. The visualization analyses for each time point were conducted independently. Read abundance was normalized by Integrative Genomics Viewer function (million per total read count). Red arrows indicate the position of predicted early, middle, and late promoter sequence *in silico* analysis.

To understand how the phage expression patterns are regulated with no change in host transcriptional levels, we examined the upstream regions of all phage genes. Grouping of promoters according to the timing of gene expression revealed similar motif signatures that are likely to be responsible for directing the expression of the early, middle, and late genes (**Figure 4**). A canonical cyanobacterial σ^70^ (SigA) recognition-like sequence was found upstream of the early genes, and comprised two palindromic 6-bp motifs, separated by 16–18 bp, located 6–8 bp upstream of the transcriptional start site (**Figure 4A**). This is consistent with current knowledge on phage early transcription, as early phage gene expression is usually regulated by the host core transcriptional machinery, and hence, phage early promoters are expected to resemble host σ^70^ promoters (Miller et al., 2003; Roucourt and Lavigne, 2009). Similarly, the promoters of the Ma-LMM01 middle genes were characterized by the SigA recognition site, suggesting that these genes were also transcribed using the core host transcriptional machinery (**Figure 4B**). Finally, although late-gene promoters showed a distinct motif, it was still very similar to the SigA recognition motif as well as early- and middle-gene promoters (**Figure 4C**). Late transcription of T4-like phages is generally independent of the host σ^70^, and is instead mediated by a phage-encoded σ factor. Hence, phage late-gene promoters are expected to resemble T4 late promoters (Miller et al., 2003; Roucourt and Lavigne, 2009; Doron et al., 2016). Therefore, in contrast to current knowledge on phage transcriptional regulation, these results suggest that all phases of Ma-LMM01 gene transcription are dependent on host σ^70^. Overall, we defined four early gene promoters, 97 middle-gene promoters, and four late-gene promoters upstream of phage genes. Transcriptomic read mapping pattern of viral genes supported the results of promoter prediction in each expression classes (**Figure 5**). Furthermore, we determined that the most plausible Ma-LMM01 early-, middle-, and late-gene promoter sequences were TAGNNNN_16-18_YATANT, TTNNNNN_10-25_TANNNT, and WTNNANN_16-22_TATTMT, respectively (**Figure 4**).

## Discussion

This study is the first report on both the transcriptional profile of cyanophage Ma-LMM01 during host infection, and the whole transcriptional response of *M. aeruginosa* to the phage infection. Our results revealed that Ma-LMM01 infection did not affect the transcriptional levels of most host genes (99.83%) at any point during the infection process (**Figure 2**). We previously investigated transcriptional alterations of host genes involved in cellular processes during Ma-LMM01 infection (4 housekeeping genes, 7 stress response genes, 3 carbonhydrate metabolic genes, and 5 photosynthetic genes) (Honda et al., 2014). In this analysis, no remarkable change in expression levels of these genes is observed (Honda et al., 2014). Thus, unlike other marine T4-cyanophages (Clokie et al., 2006; Doron et al., 2016; Thompson et al., 2016), an incomplete switch to phage transcription was observed in the infected cells throughout the Ma-LMM01 infection process. This result suggested that Ma-LMM01-encoded proteins did not cause the changes in host transcriptional activity as seen in T4 phage (Miller et al., 2003; Roucourt and Lavigne, 2009). Ma-LMM01 lacks phage-encoded proteins that are important for switching from host to phage transcription, including AsiA (anti-σ factor), MotA (activator for middle transcription), gp33 (co-activator for late transcription), and gp55 (T4-encoded σ factor) (Yoshida et al., 2008; Roucourt and Lavigne, 2009). Therefore, these results coincide with the genomic features of Ma-LMM01.

Similar results were also obtained for the PRD1 and PRR1 phage during infection of *E. coli* K-12 (Poranen et al., 2006) and *Pseudomonas aeruginosa* PAO1 (Ravantti et al., 2008), respectively. These phages are thought to down-regulate host protein synthesis, mainly by controlling ppGpp concentration (Poranen et al., 2006) and ribosomal protein synthesis (Ravantti et al., 2008), respectively, to channel host resources for viral reproduction. However, none of the host genes involved in protein synthesis showed a significant change in expression during Ma-LMM01 infection (**Figure 2**). An infection profile that does not affect host transcriptional levels might be advantageous for Ma-LMM01 to ensure viral propagation without the induction of host defense systems. *M. aeruginosa* possesses the highest number of putative antiviral defense systems of any prokaryote or archeal species examined to date (Makarova et al., 2011), including CRISPR-Cas systems, restriction-modification systems, Toxin-Antitoxin (TA) systems, and abortive infection systems. In particular, putative TA genes are highly abundant in the genome of *M. aeruginosa* (396 TA genes of 492 total defense genes) (Makarova et al., 2011). A TA system is generally composed of a stable toxin protein and an unstable antitoxin protein or small RNA, meaning that it is essential to continuously synthesize antitoxin to neutralize the toxin (Lobato-Márquez et al., 2016). Therefore, phage-mediated host transcriptional shutoff may robustly induce programmed cell death caused by TA systems in this species. Of the distinct defense genes, the type I-D CRISPR-associated protein Cas10d/Csc3 gene showed significant change in transcriptional level during infection (**Supplementary Table S2**). In CRISPR-Cas systems, the incorporation of foreign DNA fragments into the CRISPR array, mediated by Cas1 and Cas2, first occurs in the adaptation stage. CRISPR RNAs (cRNAs), which are transcribed from the CRISPR array in the expression and interference stages, then function as guides to specifically target and cleave the nucleic acids of cognate viruses or plasmids with the aid of the Cas proteins. Multiprotein crRNA-effector complexes including Cas10d/Csc3 protein mediate the processing and interference stages of type I-D CRISPR-Cas system (Makarova et al., 2015). However, in the current study, the expression of other CRISPR-Cas system related genes were not significantly altered during infection, indicating that CRISPR-Cas systems may not be effective against Ma-LMM01 infection even though *M. aeruginosa* NIES-298 possess spacers matching for this viral genome (Kuno et al., 2012). This fact supported the hypothesis that infection profile without affecting host transcriptional levels might enable Ma-LMM01 to escape the highly abundant host defense systems during infection. Unknown non-enzymatic peptides are recently thought to be significantly important for viruses to take-over host metabolism (De Smet et al., 2016). Among numerous Ma-LMM01- genes with unknowns (Yoshida et al., 2008), 10 early genes observed in this study (**Figure 3**) may be important to repress host responses to phage infection. Host transcriptional responses in the early infection in other marine cyanophages (Lindell et al., 2007; Doron et al., 2016) were not observed in this study, which supports this idea. Another possibility is that *M. aeruginosa* may control or limit the highly abundant TA systems so as not to frequently induce programmed cell death. Also, the function of up-regulated membrane protein gene remains to be understood although bacterial cell surface-related genes are associated with blocking phage adsorption (Labrie et al., 2010).

Like other T4-like marine cyanophages, Ma-LMM01 showed three temporal expression classes: early, middle, and late (**Figure 3**). The qRT-PCR based phage expression patterns supported this result (**Supplementary Figure S3**). In addition, promoter motifs linked to each of the expression classes were similar to those of cyanobacterial primary σ factor SigA recognition-like sequences, although they differed slightly from each other (**Figure 4**). Transcription in cyanobacteria is controlled by the host RNA polymerase core enzyme in combination with heterogeneous σ factors that are assigned into groups 1 (SigA), 2 (SigB–E), and 3 (SigF–J) (Imamura and Asayama, 2009). Group 1 and 3 σ factors are essential for cell viability and survival under stress, respectively, whereas group 2 σ factors are non-essential for cell viability. These findings suggest that Ma-LMM01 does not utilize alternative σ factors but favors the primary σ factor SigA for gene expression. This hypothesis coincides with the viral genomic features and host transcriptional responses observed during Ma-LMM01 infection. However, the homogenous promoter sequences raise the question as to how Ma-LMM01 controls the three temporal expression patterns during infection. One possibility is that the slight differences among promoter sequences may contribute to distinguishing between the three promoter types as well as the host promoters (Roucourt and Lavigne, 2009). Another possibility is that early gene products (Gp054–Gp063) may be involved in the regulation of viral expression patterns during Ma-LMM01 infection. In general, phage-encoded early products usually modify the host RNA polymerase complex, and then switch viral expression classes (Roucourt and Lavigne, 2009). However, Ma-LMM01 early gene products may employ a novel mechanism to control their expression patterns, as phage gene products usually down-regulate host transcription (Roucourt and Lavigne, 2009).

In addition to the scenario that Ma-LMM01 maintained host transcriptional activity as described above, there is an alternative possibility that decomposition as well as production of host transcripts did not occur, and thereby host transcripts were apparently stable. In T4 phage, host transcription is halted shortly after infection even though viral reproduction are independent of host transcripts at all (Miller et al., 2003; Roucourt and Lavigne, 2009), which supports this scenario. In either scenario, it is possible that Ma-LMM01 encounters nutrient limitation during infection, particularly in molecules such as nucleic acids and amino acids that are required for viral reproduction. In general, bacteriophages exploit the host metabolism to establish an efficient infection cycle and redirect host cell components, including metabolic substrates and the machinery for replication, transcription, and translation, toward the production of new virions (Miller et al., 2003). In T4 phage, for example, nucleotide precursors for DNA replication are generated from host DNA degradation, and host transcription is down-regulated by the sequential modification of host transcriptional machinery (Miller et al., 2003). In addition, T4-like marine cyanophages redirect carbon flux from the Calvin cycle to the pentose phosphate pathway, maintaining host photosynthesis by using AMGs (Thompson L.R. et al., 2011; Thompson et al., 2016). However, Ma-LMM01 lacks the defined genes that are required for the acquisition of precursors for their replication and virion morphogenesis (Yoshida et al., 2008). One possibility is that viral DNA replication and virion synthesis proceed gradually during Ma-LMM01 infection using the remaining precursors inside the infected cells. This hypothesis is supported by the observations that middle-gene transcriptional levels remained elevated up to 3–6 h post-infection, and late-gene transcriptional levels increased gradually during the latent period (**Figure 3**). Also, *M. aeruginosa* has a larger genome (4.92 Mb) than marine cyanobacteria such as *Prochlorococcus* and *Synechococcus* (1.64–2.86 Mb) (Scanlan et al., 2009). This suggests that unlike in other marine cyanobacteria, nucleotide precursors are highly abundant in *Microcystis* cells, and can therefore be exploited by Ma-LMM01 for its own DNA replication. Furthermore, phage-encoded NblA may provide amino acid precursors that are required for viral protein synthesis during Ma-LMM01 infection. The degradation of phycobilisomes, catalyzed by NblA, is thought to provide a pool of resources that can be reused by cyanobacteria during nutrient limitation (Collier and Grossman, 1994). In addition, phage-encoded NblA is active in *Planktothrix* phage PaV-LD, and degrades the host phycobilisomes (Gao et al., 2012). Also, we previously reported that Ma-LMM01-encoded *nblA* is expressed in the infected culture and that host phycobilisomes are degraded during Ma-LMM01 infection (Yoshida et al., 2008). These findings suggest that Ma-LMM01-encoded NblA contributes to sustaining amino acid pools to prevent nutrient limitation. Phage-encoded NblA and host heat-shock proteins such as GroES and hspA are thought to contribute the maintenance of photosynthesis activity during Ma-LMM01 infection (Honda et al., 2014). According to this idea, transcriptional levels of *GroES, GroEL* and heat-shock protein gene significantly increased at 6 h post-infection (**Supplementary Table S2**). Thus, Ma-LMM01 may provide the precursors and energy for their reproduction by maintaining photosynthetic apparatus and degradation of phycobilisome. In addition, T4 phage halts phage development until appropriate nutrients become available in the stationary phase *E. coli* cells although DNA replication is completed (Bryan et al., 2016). Similarly, T4-like marine cyanophage P-SSM2 responds to phosphate limited conditions and maintains the host phosphate uptake rate during infection by controlling host PhoR/PhoB two-component signal transduction system (Zeng and Chisholm, 2012; Lin et al., 2016). Ma-LMM01 which encounters nutrient limitation during infection may possess similar mechanisms that adjust infection process and host physical states in response to cellular conditions as seen in these phages. Another possibility is that Ma-LMM01 may control host metabolism in translational levels to provide the precursors such as nucleotides and amino acids for their reproduction. Indeed, cellular adaptation for the production of phage progeny is thought to be more active at the translational or posttranslational level in *Lactococcus lactis* phage Tuc2009 and c2 (Ainsworth et al., 2013) and *Pseudomonas aeruginosa* phage LUZ19 (Lavigne et al., 2013), in which the minimal transcriptional response is observed during infection. Translational dynamics during Ma-LMM01 infection will help us to further understand the viral impacts on host physiology.

## Conclusion

Ma-LMM01 employs an infection program in the apparently stable host transcriptional levels, and uses the host core σ factor SigA while avoiding host defense systems. This type of infection is a novel example of adaptation based on host defense systems to ensure efficient viral reproduction, and differs from that seen in other marine T4-like cyanophages. Future work is needed to explore whether other types of cyanophage infecting *M. aeruginosa* show the same transcriptome dynamics and infection program.

## Author Contributions

DM performed the experiment and prepared the manuscript. SK and YS contributed to manuscript discussion and revision. TY contributed to the experimental design, result discussion, manuscript revision and overall support of this study.

## Conflict of Interest Statement

The authors declare that the research was conducted in the absence of any commercial or financial relationships that could be construed as a potential conflict of interest.

## Footnotes

1 http://www.nies.go.jp

2 http://softberry.com

## Supplementary Material

FIGURE S1 Rarefaction curves and chao1 indices for each library. Rarefaction curves of host **(A)** and viral **(B)** reads were generated from library at each time point. The chao1 indices were also used to evaluate each library.

FIGURE S2 *rnpB* transcript levels during infection. Transcript levels of *rnpB* from *Microcystis aeruginosa* NIES-298 during the 8-h latent period of infection by the Ma-LMM01 phage as determined by quantitative real-time polymerase chain reaction analysis. The copy number of *rnpB* at each time point was normalized per nanogram of total RNA. Results corresponding to the infected culture are shown in red, while the uninfected control culture results are shown in blue. Three technical replicates were carried out for each biological replicate. This result was verified by transcriptional dynamics from RNA-seq data that total reads at each time point were mapped to *rnpB* sequence (shown in black). Transcript count for *rnpB* was normalized as FPKM.

FIGURE S3 Quantitative real-time polymerase chain reaction (qRT-PCR) validation of RNA-seq results. The RNA-seq results were verified using qRT-PCR analysis of host *sigA* and viral *gp091* gene expression during infection **(A)**. Temporal expression classes were also verified with putative early (*gp054, gp062*), middle (*gp087, gp134*), and late (*gp087, gp091*) genes respectively **(B)**. Transcript levels for each gene were normalized to host *rnpB* transcript levels.

FIGURE S4 Heat map of Ma-LMM01 genes during infection. Genes are listed in order on the Ma-LMM01 genome. The color gradient indicates gene transcripts unchanged (green) or enriched (red).

FIGURE S5 Jaccard similarity coefficients. The Jaccard coefficient was used as a stability measure to determine the most stable number of clusters obtained by hierarchical clustering analysis of the phage genes. Individual histograms show the distribution of the Jaccard coefficients for 1000 subsets of genes when 70% of the genes are randomly selected for each subset. Each histogram displays the distribution of Jaccard coefficients for a different number of clusters, k (*k* = 2 to *k* = 7). Clustering was considered stable when the majority of Jaccard values were close or equal to one.

FIGURE S6 Genome organization of phage early, middle, and late genes. Log_2_ fold changes in expression of phage genes during infection of *Microcystis aeruginosa* NIES-298 at 1 h (green), 3 h (blue), and 6 h (red) post-infection.

TABLE S1 Summary of sequencing data generated in this study.

TABLE S2 Host genes with a significant change in transcript levels during phage infection. Log_2_ fold changes (FC) in gene expression of infected versus control cultures with time (h) following infection. Positive values indicate an increase in transcript levels during infection, with significant differences in Log_2_ FC (*P* < 0.05) shown in red.

TABLE S3 Summary of viral gene expression patterns, promoters, and expression data.
