# Supplementary figures and images for: Transcriptome Analysis of a Bloom-Forming Cyanobacterium Microcystis aeruginosa during Ma-LMM01 Phage Infection
Source: Front Microbiol. 2018 Jan 19;9:2. doi: 10.3389/fmicb.2018.00002 (PMC5780444; doi:10.3389/fmicb.2018.00002)

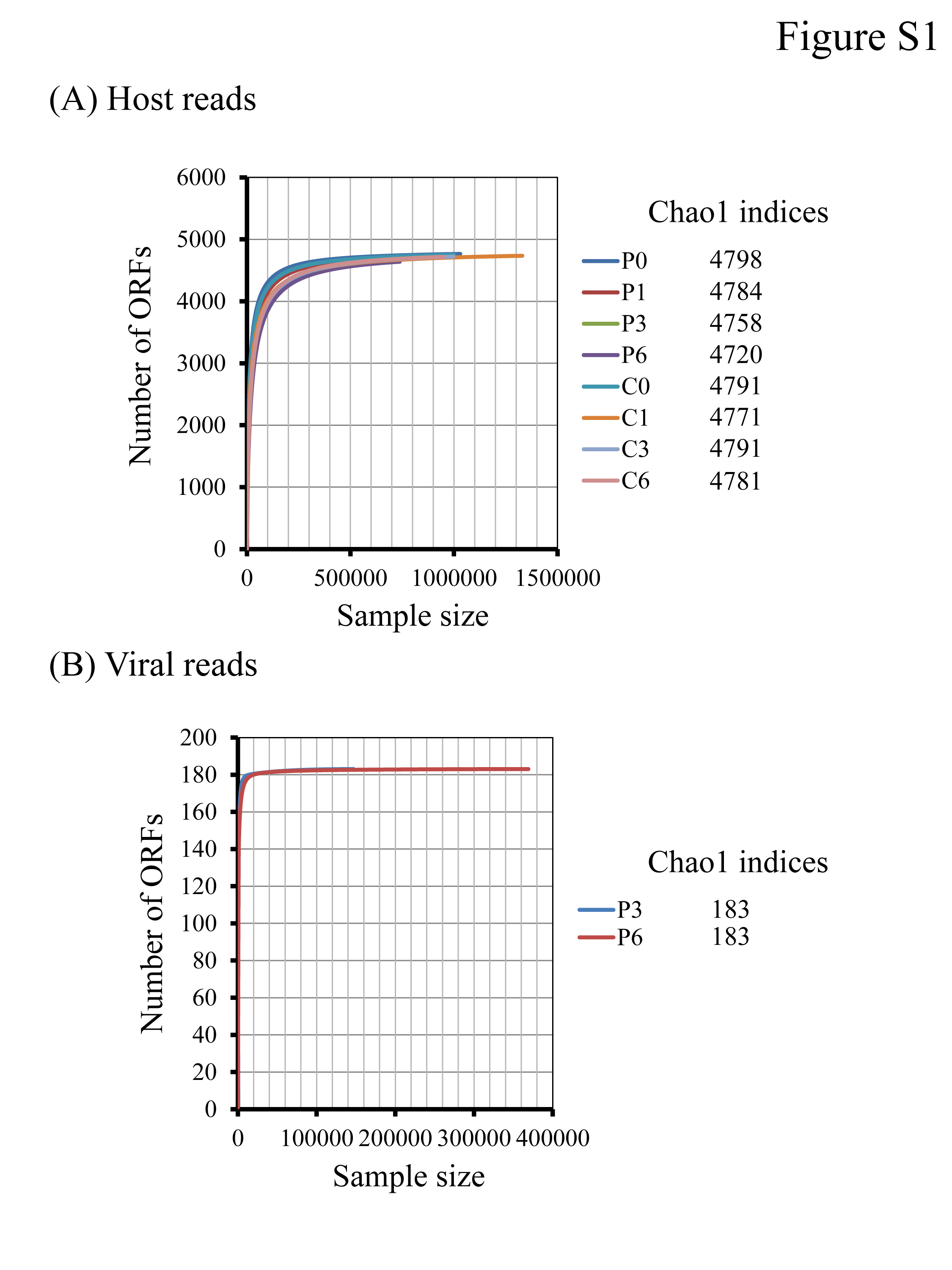

Supplement: FIGURE S1 — Rarefaction curves and chao1 indices for each library. Rarefaction curves of host (A) and viral (B) reads were generated from library at each time point. The chao1 indices were also used to evaluate each library. [file Image_1.TIF]

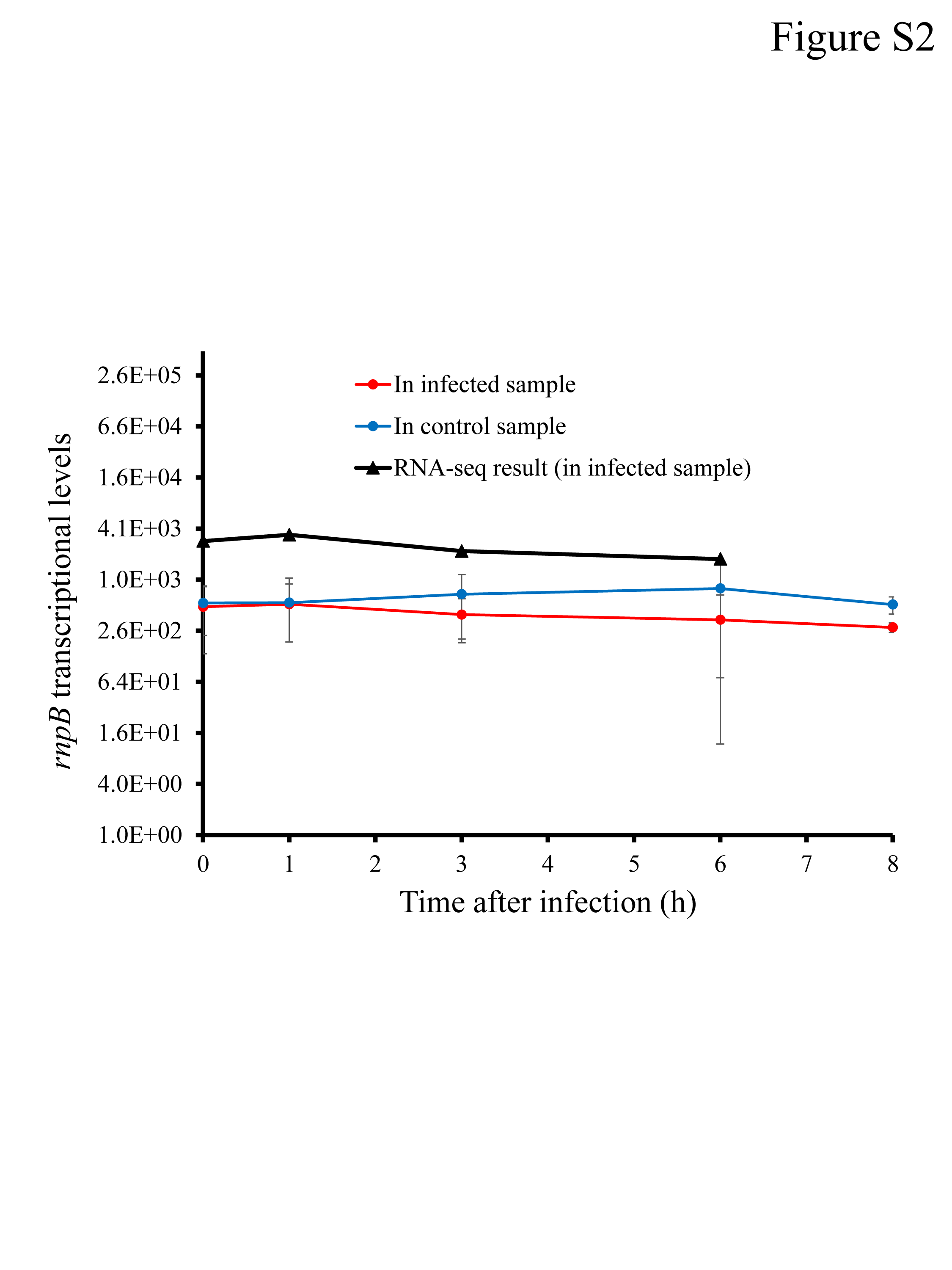

Supplement: FIGURE S2 — rnpB transcript levels during infection. Transcript levels of rnpB from Microcystis aeruginosa NIES-298 during the 8-h latent period of infection by the Ma-LMM01 phage as determined by quantitative real-time polymerase chain reaction analysis. The copy number of rnpB at each time point was normalized per nanogram of total RNA. Results corresponding to the infected culture are shown in red, while the uninfected control culture results are shown in blue. Three technical replicates were carried out for each biological replicate. This result was verified by transcriptional dynamics from RNA-seq data that total reads at each time point were mapped to rnpB sequence (shown in black). Transcript count for rnpB was normalized as FPKM. [file Image_2.TIF]

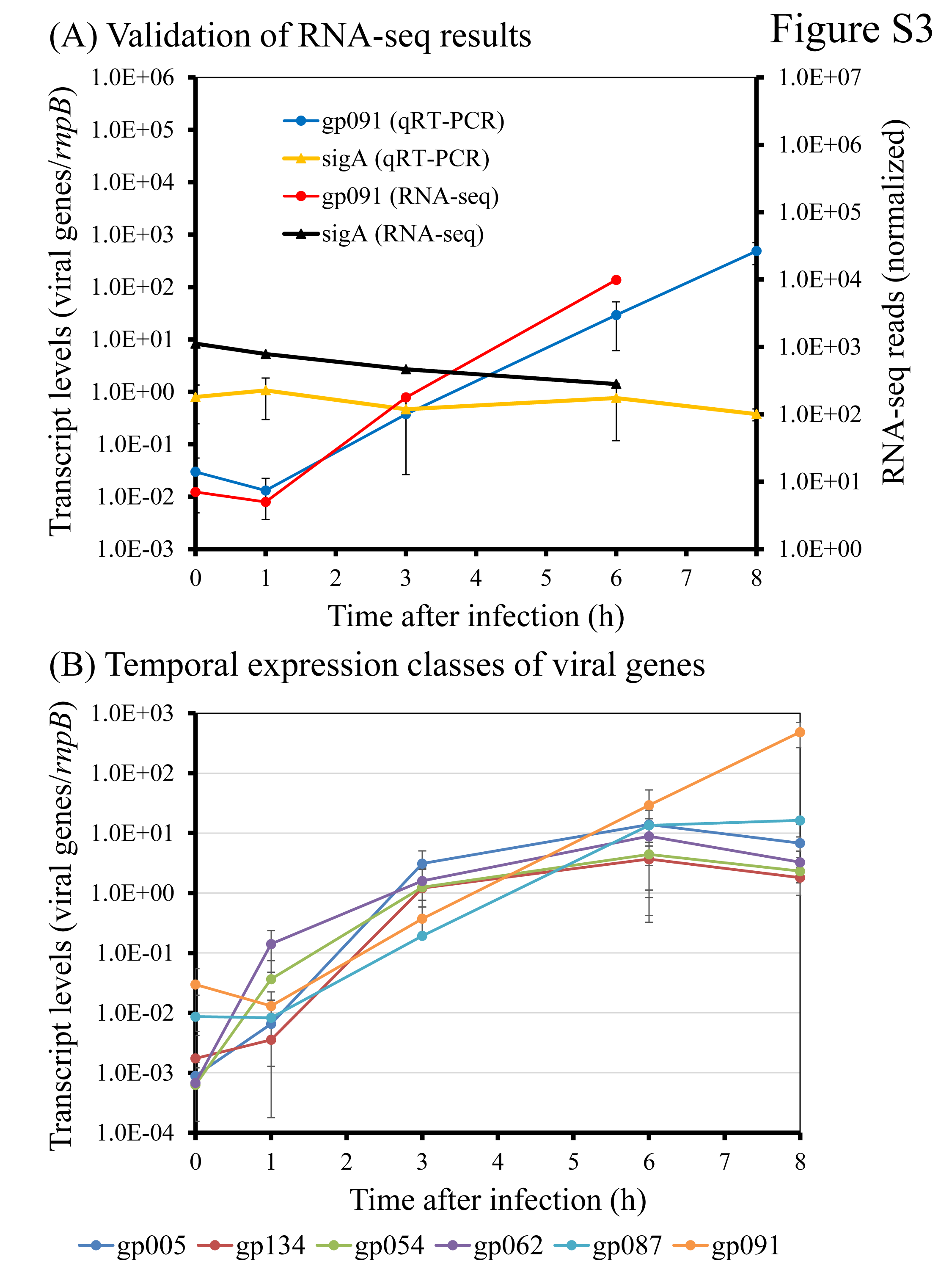

Supplement: FIGURE S3 — Quantitative real-time polymerase chain reaction (qRT-PCR) validation of RNA-seq results. The RNA-seq results were verified using qRT-PCR analysis of host sigA and viral gp091 gene expression during infection (A). Temporal expression classes were also verified with putative early (gp054, gp062), middle (gp087, gp134), and late (gp087, gp091) genes respectively (B). Transcript levels for each gene were normalized to host rnpB transcript levels. [file Image_3.TIF]

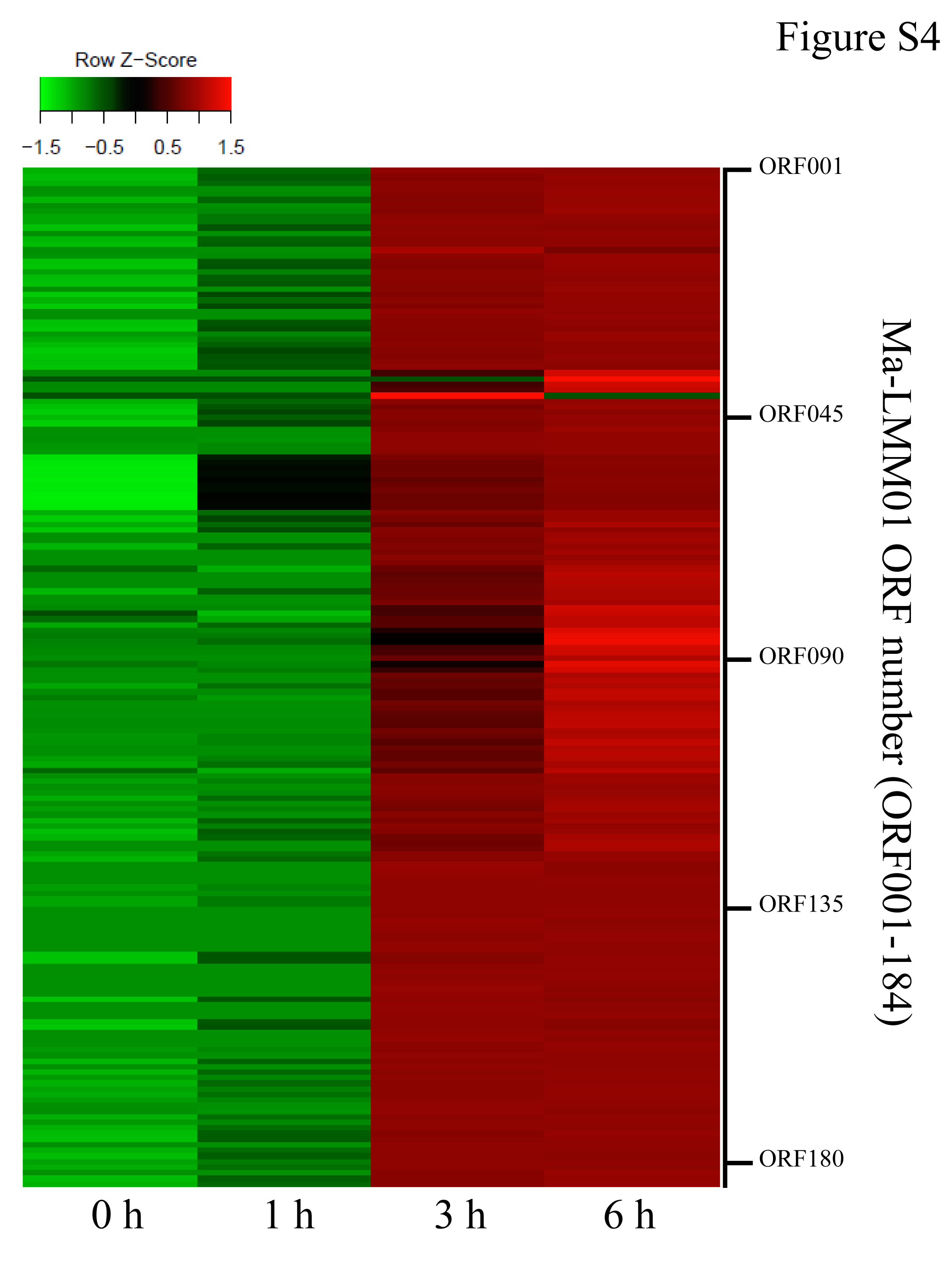

Supplement: FIGURE S4 — Heat map of Ma-LMM01 genes during infection. Genes are listed in order on the Ma-LMM01 genome. The color gradient indicates gene transcripts unchanged (green) or enriched (red). [file Image_4.TIF]

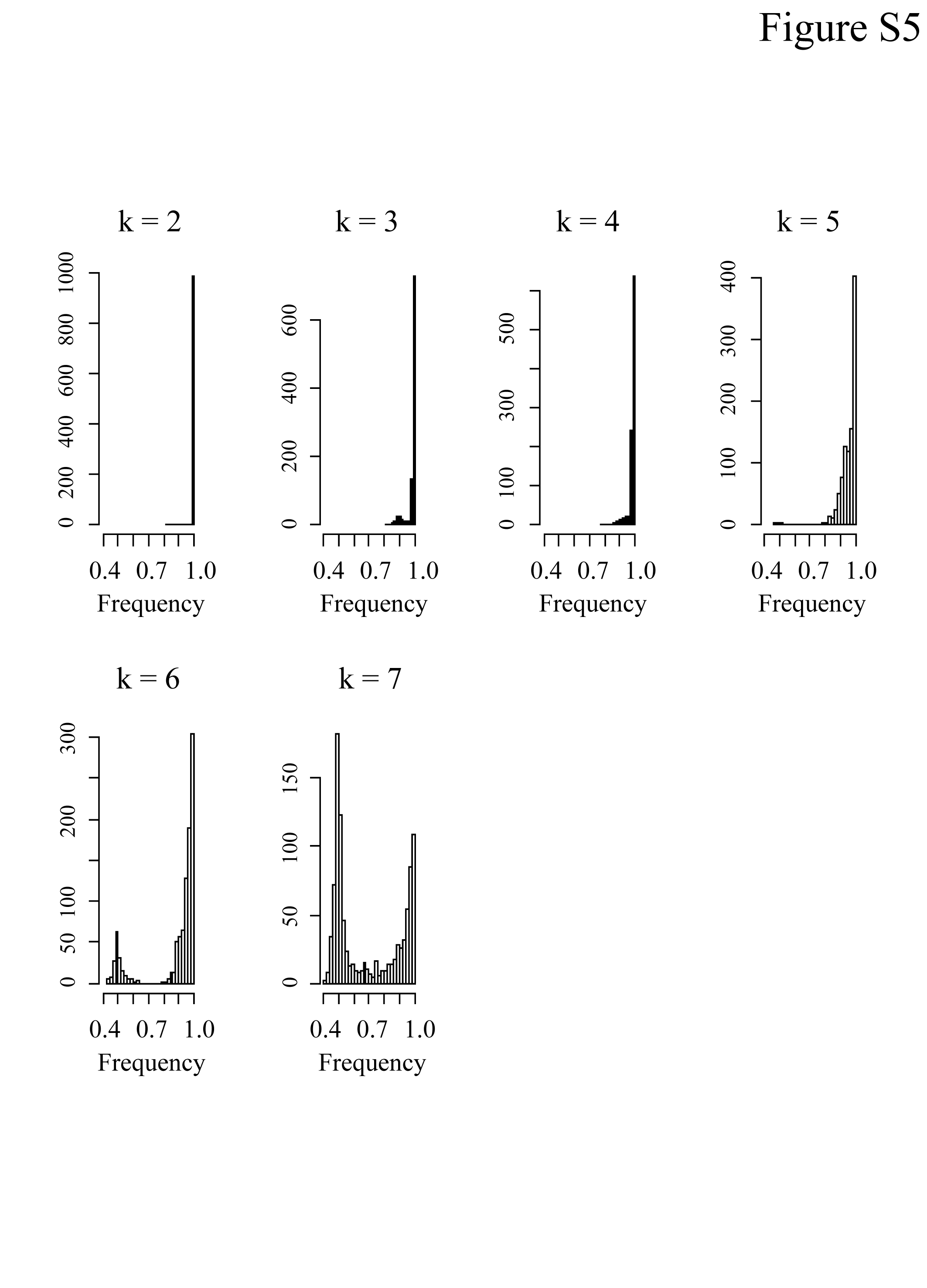

Supplement: FIGURE S5 — Jaccard similarity coefficients. The Jaccard coefficient was used as a stability measure to determine the most stable number of clusters obtained by hierarchical clustering analysis of the phage genes. Individual histograms show the distribution of the Jaccard coefficients for 1000 subsets of genes when 70% of the genes are randomly selected for each subset. Each histogram displays the distribution of Jaccard coefficients for a different number of clusters, k (k = 2 to k = 7). Clustering was considered stable when the majority of Jaccard values were close or equal to one. [file Image_5.TIF]

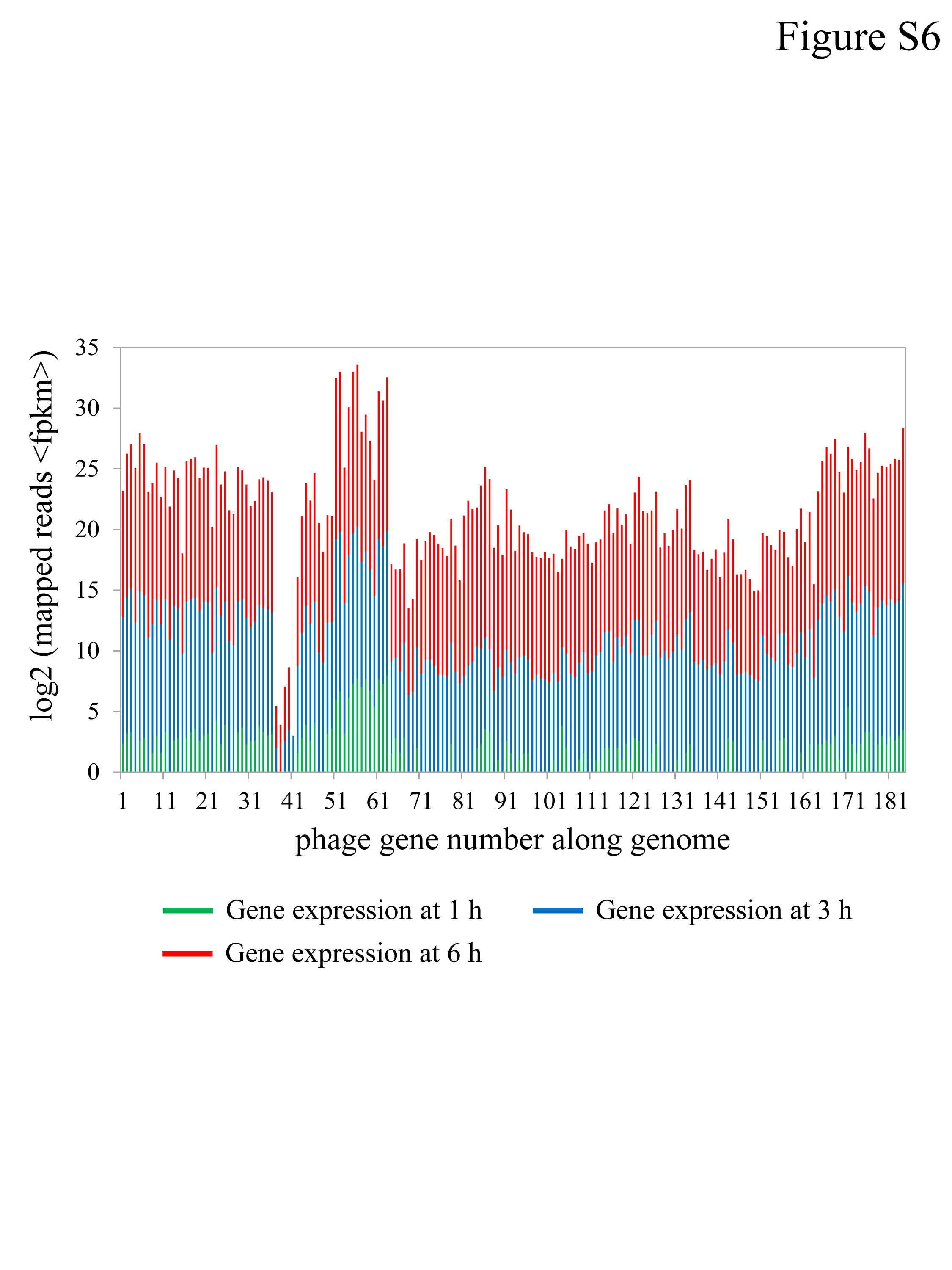

Supplement: FIGURE S6 — Genome organization of phage early, middle, and late genes. Log2 fold changes in expression of phage genes during infection of Microcystis aeruginosa NIES-298 at 1 h (green), 3 h (blue), and 6 h (red) post-infection. [file Image_6.TIF]
